# Ex vivo expanded human cord blood-derived hematopoietic progenitor cells induce lung growth and alveolarization in injured newborn lungs

**DOI:** 10.1186/1465-9921-14-37

**Published:** 2013-03-23

**Authors:** Quanfu Mao, Sharon Chu, Sailaja Ghanta, James F Padbury, Monique E De Paepe

**Affiliations:** 1 Department of Pathology, Women and Infants Hospital, Providence, RI, USA; 2 Department of Pediatrics, Providence, RI, USA; 3 Department of Pathology and Laboratory Medicine, 101 Dudley Street, Providence, RI, 02905, USA; 4 Department of Pediatrics, Alpert Medical School of Brown University, Providence, RI, 02905, USA

**Keywords:** Alveolar type II cell, Bronchopulmonary dysplasia, Dexamethasone, Stem cell, Regeneration

## Abstract

**Background:**

We investigated the capacity of expanded cord blood-derived CD34+ hematopoietic progenitor cells to undergo respiratory epithelial differentiation *ex vivo*, and to engraft and attenuate alveolar disruption in injured newborn murine lungs *in vivo*.

**Methods:**

Respiratory epithelial differentiation was studied in CD34+ cells expanded in the presence of growth factors and cytokines (“basic” medium), in one group supplemented with dexamethasone (“DEX”). Expanded or freshly isolated CD34+ cells were inoculated intranasally in newborn mice with apoptosis-induced lung injury. Pulmonary engraftment, lung growth and alveolarization were studied at 8 weeks post-inoculation.

**Results:**

SP-C mRNA expression was seen in 2/7 CD34+ cell isolates expanded in basic media and in 6/7 isolates expanded in DEX, associated with cytoplasmic SP-C immunoreactivity and ultrastructural features suggestive of type II cell-like differentiation. Administration of expanding CD34+ cells was associated with increased lung growth and, in animals treated with DEX-exposed cells, enhanced alveolar septation. Freshly isolated CD34+ cells had no effect of lung growth or remodeling. Lungs of animals treated with expanded CD34+ cells contained intraalveolar aggregates of replicating alu-FISH-positive mononuclear cells, whereas epithelial engraftment was extremely rare.

**Conclusion:**

Expanded cord blood CD34+ cells can induce lung growth and alveolarization in injured newborn lungs. These growth-promoting effects may be linked to paracrine or immunomodulatory effects of persistent cord blood-derived mononuclear cells, as expanded cells showed limited respiratory epithelial transdifferentiation.

## Background

Premature infants treated with supplemental oxygen and mechanical ventilation are at risk for bronchopulmonary dysplasia (BPD), or chronic lung disease of the preterm newborn, a complex and multifactorial condition characterized by an arrest of alveolar development [1-4]. Although increased use of exogenous surfactant and antenatal steroids, improved ventilatory strategies, and changes in neonatal intensive care have modified its phenotype, BPD continues to be a significant health problem for the increasing number of surviving extremely premature infants.

The role of stem cell therapy as an alternative or complimentary approach for treatment or prevention of chronic lung disease of the newborn remains largely undetermined (reviewed in [5,6]). Cord blood might represent an attractive source of stem cells for regenerative therapy in newborns. Human umbilical cord blood stem cells can be collected at no risk to the donor, have low immune reactivity and low inherent pathogen transmission, and are not subject to the social and political controversy associated with embryonic stem cells. Cord blood stem cells are particularly attractive in the newborn context where, ideally, the infant’s own cord blood-derived stem cells could be used as an autologous transplant in a contained system, thus limiting the risk of infection or rejection. Cord blood stem cell harvesting is expected to be most applicable to newborns of more than 30-32 weeks’ gestational age; it may be impossible to obtain sufficient amounts of cord blood at younger ages due to the small size of cord and placental/umbilical vessels.

Among the various types of stem cells that can be procured from umbilical cord blood (CB), CD34+ hematopoietic progenitor cells are relatively uniformly characterized, easily isolated, and have an excellent and long-standing safety record after decades of use in clinical bone marrow transplantation. We previously demonstrated that freshly isolated human cord blood-derived (CB) CD34+ hematopoietic progenitor cells are capable of long term pulmonary engraftment, replication, clonal expansion, and differentiation to alveolar type II cell-like cells by apparent fusion-independent mechanisms following intranasal/intrapulmonary inoculation in newborn mice [7]. Furthermore, we determined that the engrafted cord blood-derived transdifferentiated alveolar type II cells function as progenitor cells of the distal respiratory unit, similar to endogenous type II cells [7]. This proof-of-concept pilot study allowed us to establish the *in vivo* transdifferentiation potential of CB-CD34+ cells. However, the small graft size used in that study (1-2 x 10^5^ cells/pup) did not allow definitive assessment of the potential functional capacity of cord blood stem cells to prevent or attenuate alveolar disruption in injured newborn lungs.

Successful translation of alveolar epithelial cell therapy to preclinical or clinical contexts may depend on our capacity to deliver stem or progenitor cells in sufficient quantity to achieve functional benefits. The main strategies to augment graft size consist of either pooling of stem cells from multiple donors or expansion of stem cells from a single donor. While both methods have merit, and may well be complimentary, *ex vivo* expansion seems a particularly attractive technique in newborn infants as it might allow transplantation of autologous cord blood-derived cells in a fully contained system.

The aim of this study was to test the hypothesis that CB-CD34+ cells, expanded *ex vivo* in conditions promoting respiratory epithelial differentiation, retain their capacity for pulmonary engraftment and respiratory epithelial differentiation and can promote lung growth and alveolarization in injured newborn lungs *in vivo*. For *ex vivo* induction of respiratory epithelial differentiation, CB-CD34+ cells were exposed to various factors known to promote lung maturation, differentiation, growth, and/or repair, including retinoic acid, keratinocyte growth factor, GM-CSF and dexamethasone [8-17]. As in previous studies [7,18], we chose the intranasal/intrapulmonary, rather than systemic route of administration for delivery of stem cells. The direct intrapulmonary delivery of stem cells may represent a biologically more sound strategy for restoration of the respiratory epithelium [18]. Furthermore, as many preterm infants are intubated, intrapulmonary delivery via the endotracheal tube is clinically relevant and within the scope of the current practice of administration of exogenous surfactant and antioxidants.

As model of neonatal lung injury, we used our previously described conditional respiratory epithelium-specific Fas-ligand (FasL) overexpressing transgenic mouse [19,20]. When FasL-mediated respiratory epithelial cell death is targeted to the perinatal period, this novel and versatile transgenic mouse model provides replication of both the early apoptotic injury and subsequent alveolar simplification typical of preterm infants with BPD [19,20].

## Methods

### Isolation and culture of CB-CD34+ cells

Umbilical cord blood (CB) CD34+ cells were isolated from uncomplicated full-term cesarean deliveries at Women and Infants Hospital according to protocols approved by the Institutional Review Board, as described [7]. In accordance with our previous experience, on average about 1.5 x 10^6^ CB-CD34+ cells were harvested per placenta. CD34+ cell purity was greater than 95% and viability greater than 92% following density gradient centrifugation and immunomagnetic (MACS) sorting.

Freshly isolated CB-CD34+ cells were cultured in liquid suspension according to a two-step expansion and differentiation protocol. Initially, enriched CB-CD34+ cells were incubated in StemPro-34 Serum-Free Medium (SFM) (Invitrogen, Carlsbad, CA) supplemented with the following human recombinant factors: stem cell factor (SCF, 100 ng/ml), IL-3 (50 ng/ml) and GM-CSF (25 ng/ml) (all from Miltenyi Biotec) (“expansion” medium). Cells were incubated in 25 cm^3^ vented tissue culture flasks in 5-10 mL expansion media in a fully humidified atmosphere of 5% CO_2_ at 37°C.

After 72 hours of culture in StemPro-34 SFM expansion medium, cells were incubated in two types of culture media aimed at inducing respiratory epithelial differentiation (“differentiation” media). The following differentiation media were assessed: StemPro-34 SFM medium, supplemented with retinoic acid (RA, 0.01 μM) (Sigma, St. Louis, MO) and keratinocyte growth factor (KGF, 0.01 μM, Sigma) in addition to the human recombinant factors SCF, IL-3 and GM-CSF (“basic” medium) and StemPro basic medium supplemented with dexamethasone (DEX) (Sigma) at concentrations ranging between 10^-5^ and 10^-7^ M (in 0.01% DMSO) (“DEX” medium). StemPro basic medium with 0.01% DMSO (“DMSO” medium) served as additional control for the dexamethasone cultures. At three-day intervals, one-half volume fresh media was added to the cultures, at which time half of the medium and cells from each flask were collected for analyses.

### Analysis of expansion kinetics of cultured CB-CD34+ cells

Aliquots of cultured CB-CD34+ cells were taken at 3-day intervals and stained with 0.4% Trypan Blue (vital dye) (Life Technologies, Grand Island, NY). The total number of live cells (unstained) was counted using a hemocytometer. The growth rate was defined as the total cell number at a particular time point divided by the cell number at the preceding time point. The expansion index was determined by dividing the total cell number at a particular time point by the total cell number on day 0 and reflects the degree of amplification of the cell population. In three experiments, the fraction of CD34+ cells at selected points was analyzed by flow cytometry using FITC-labeled anti-CD34 antibodies (Miltenyi Biotec, Cambridge, MA).

### Analysis of respiratory epithelial differentiation of cultured CB-CD34+ cells

The cellular morphology of cultured CB-CD34+ cells at various time intervals after isolation was studied by phase contrast optics and light microscopy of Giemsa-Wright-stained cytospin preparations. Cytospin preparations from selected time points were subjected to anti-surfactant protein-C (SP-C) immunofluorescence analysis, using a polyclonal anti-SP-C antibody (Abcam) according to methods previously described [21]. In addition, CD34+ isolates from selected time points were studied by transmission electron microscopy. For ultrastructural studies, cell pellets were fixed with 1.25% glutaraldehyde in 0.15 mol/L sodium cacodylate buffer, postfixed with 1% osmium tetroxide, and dehydrated through a graded ethanol series. Ultrathin sections were stained with uranyl acetate/lead citrate and viewed using a Philips 300 electron microscope (Philips, Research, Eindhoven, the Netherlands).

Respiratory epithelial gene expression in expanded CD34+ cells was estimated by semi-quantitative RT-PCR analysis of expression of SP-C as marker gene of alveolar type II cells. Total cellular RNA was extracted from CB-CD34+ cell lysates at various time points using Trizol reagent (Invitrogen) and purified using the RNeasy MinElute Cleanup kit (Qiagen, Valencia, CA). Total RNA (1 μg) was reverse-transcribed using the SuperScript III First-Strand Synthesis System (Invitrogen) according to the manufacturer’s protocol. Surfactant protein C (SP-C) and glyceraldehyde phosphate dehydrogenase (GAPDH, housekeeping gene) were amplified by polymerase chain reaction (PCR). The following primer sequences were used: SP-C: F: TGG TCC TCA TCG TCG TGG TGA TTG; R: CCT GCA GAG AGC ATT CCA TCT GGA AG (product size: 327 bp) and GAPDH: F: CCC TTC ATT GAC CTC AAC TAC AT; R: ACG ATA CCA AAG TTG TCA TGG AT (product size: 407 bp).

### Animal husbandry and tissue processing

The previously described lung-specific FasL overexpressing transgenic mouse [19,20] was used as model for neonatal lung injury/BPD. This model is based on a tetracycline-dependent tet-on overexpression system to achieve time-specific FasL transgene expression in the respiratory epithelium [19]. Transgenic (tetOp)_7_-FasL mice (“responder line”) were crossed with CCSP-rtTA mice (“activator line”) (kindly provided by Dr. J. Whitsett, University of Cincinnati, OH) [22] to yield a mixed offspring of double transgenic (CCSP-rtTA+/(tetOp)_7_-FasL+) and single transgenic (CCSP-rtTA+/(tetOp)_7_-FasL-) littermates. Upon exposure to the tetracycline analogue, doxycycline (Dox), double transgenic mice (CCSP+/FasL+) exhibit marked pulmonary apoptosis, resulting in BPD-like alveolar disruption [19]. The transgenic animals are generated in a FVB/N genetic background and have an intact immune system.

Dox (0.01 mg/ml) was added to the drinking water of pregnant and/or nursing dams from embryonal day 14 (E14) to postnatal day 3 (P3; postnatal day P1 = day of birth). Pups were genotyped at P2. Only double transgenic CCSP+/FasL + animals were used for these experiments. The animals were sacrificed at post-inoculation week 8 by pentobarbital overdose. Lungs were processed as described [19]. All animal experiments were conducted in accordance with institutional guidelines for the care and use of laboratory animals.

### Intranasal administration of CB-CD34+ cells to newborn mice

To determine the effects of *ex vivo* expansion on the engraftment, differentiation and regenerative capacity of CB-CD34+ cells, cultured cells were collected after 4-6 days of culture in differentiation media (i.e. 7 to 9 days after isolation) for administration to Dox-treated CCSP+/FasL + newborn mice with lung injury. At this time point, the cell number was doubling, while still approximately 60% of cells had a CD34+ progenitor cell immunophenotype. To prevent or minimize exposure of the animals to DEX and other factors used during the *ex vivo* expansion/differentiation, the cultured cells were resuspended in PBS after collection. At postnatal day 5 (P5), expanded CB-CD34+ cells from a single donor (1 × 10^6^ cells/pup) were delivered to Dox-treateddouble transgenic CCSP+/FasL + pups by intranasal administration, as previously described [18]. Experimental animals received CD34+ cells from a single donor expanded in basic medium or expanded in DEX medium (basic medium supplemented with 10^-6^ M DEX). Animals treated with expanded cells received 1 × 10^6^ cells per pup. Control animals received freshly isolated CD34+ cells (0.5 × 10^6^ cells/pup) or equal volume phosphate-buffered saline (PBS) vehicle buffer. Intranasal inoculation was performed at postnatal day 5 (P5) as this time point is characterized by marked alveolar epithelial cell apoptotic injury and remodeling in Dox-treated double transgenic CCSP+/FasL + mice.

### Analysis of engraftment and cell fate of expanded CB-CD34+ cells in lungs of newborn mice

Engraftment of cord blood-derived cells was assessed at 8 weeks post- inoculation. The presence of CB-CD34+ cells or their progeny was studied by fluorescent in situ hybridization (FISH) analysis using human-specific alu probes and analyzed by confocal microscopy, as previously described [7]. The proliferative activity of engrafted cord blood-derived cells was assessed by combining human alu-FISH analysis with anti-Ki67 immunohistochemistry [7].

In the absence of human-specific immunohistochemical markers of alveolar type II cells, differentiation of CB-CD34+ cells to alveolar type II cells was assessed by combining anti-human cytokeratin staining (used as marker of human, i.e. cord blood-derived epithelial cells) with anti-prosurfactant protein-C (SP-C) (used as alveolar type II cell marker) (ab28744, Abcam Inc., Cambridge, MA) , as previously described [7]. In addition to standard epifluorescence microscopy, the sections were viewed by confocal microscopy and slice or three-dimensional volume reconstruction and projections were generated to ascertain the veracity of co-localization phenomena, as described [18]. To study leukocyte differentiation of donor-derived cells, sections were studied by immunoperoxidase method using a human-specific anti-CD45 antibody (DAKO).

### Analysis of effect of expanded CB-CD34+ cells on lung growth and alveolarization

Morphometric assessment of growth of peripheral air-exchanging lung parenchyma and contribution of the various lung compartments (airspace versus parenchyma) to the total lung volume was performed using stereological volumetric techniques, as previously described [23,24]. The inflated lung volume, V(lu), was determined according to the Archimedes principle [25]. The areal density of air-exchanging parenchyma, A_A_(ae/lu), was determined by point-counting based on computer-assisted image analysis. The total volume of air-exchanging parenchyma, V(ae), was calculated by multiplying A_A_(ae/lu) by V(lu). Alveolarization was quantified by computer-assisted histomorphometric analysis of the mean cord length (MCL), as described [19]. All morphometric assessments were made on coded slides by a single observer who was unaware of the experimental condition of the animal analyzed.

### Data analysis

Values are expressed as mean ± standard deviation (SD). The significance of differences between groups was determined with the unpaired Student’s *t*-test or ANOVA with post-hoc Scheffe test where indicated. The significance level was set at *P* < 0.05.

## Results

### Expansion kinetics of cultured CB-CD34+ cells

**Figure 1:**
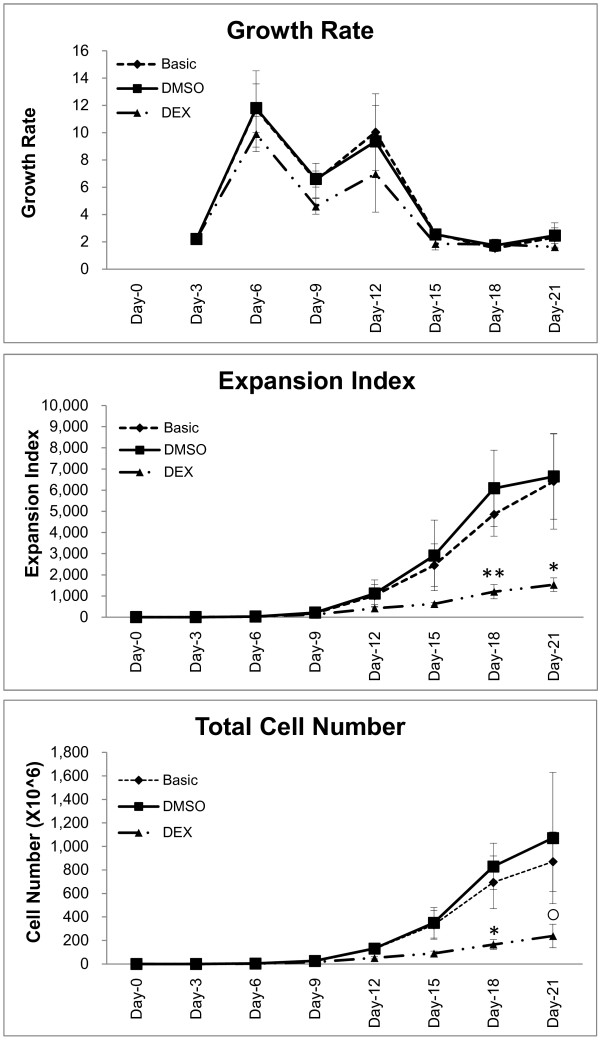
**Expansion kinetics of cultured CB-CD34+ cells.** Time course expansion of nucleated cells in basic medium, DEX medium, or DMSO medium. Data are mean ± SD obtained from three experiments performed in triplicate. *: *P* < 0.05; **: *P* < 0.02; º: *P* = 0.08 versus basic medium.

Enriched CD34+ cells were cultured in expansion media for three days, followed by incubation in media aimed at inducing respiratory epithelial differentiation (“basic” and “DEX”). The cell growth rate, defined as cell number at any given time point divided by the cell number at the immediately preceding time point, was highest in the first 2 weeks of culture for all groups and tended to be higher for cells cultured in basic medium than for cells cultured in DEX media (10^-5^ M in 0.1% DMSO) (Figure 1). During the 21-day culture period, CD34+ cells in basic medium expanded on average more than 6,000-fold, whereas expansion rates were significantly three-fold lower for cells incubated with DEX (Figure 1). The expansion kinetics of DMSO-exposed cells were comparable to those of cells incubated in basic medium (Figure 1). As expected, expansion of CB-CD34+ cells was associated with a progressive loss of the CD34+ immunophenotype. The fraction of CD34-positive cells decreased from >95% on day 0 to 90 ± 2% on day 6, 36 ± 3% on day 9, and 8 ± 3% on day 12, as determined by flow cytometry of 3 isolates expanded in DEX media.

### Morphology of expanded CB-CD34+ cells

**Figure 2:**
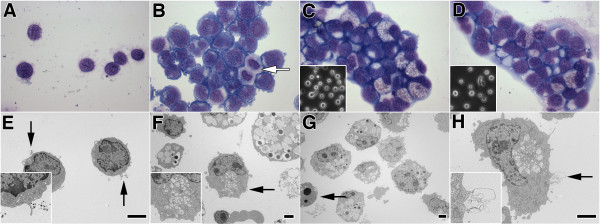
**Morphology of expanded CB-CD34+ cells. A-D.** Cytospin preparations of cultured CB-CD34+ cells. **A**. Day 0. Cells appear lymphocyte-like and uniform in size and shape. **B**. Day 3; basic medium. Amount of cytoplasm has increased. Mitotic activity is noted (arrow). **C**-**D**. Day 15; basic (**C**) or DEX (**D**) medium (insets: corresponding phase contrast microscopy images). Cells are larger and more heterogeneous. Many cells display a vacuolated cytoplasm with granular eosinophilic or basophilic cytoplasm. **A**-**D**: Giemsa-Wright stain; original magnification X 1,000. **E-H.** Ultrastructural appearance of expanded CB-CD34+ cells. **E**. Day 0. Relatively small freshly isolated cells with sparse cytoplasm and scant organelles. Electron dense beads are noted (incidental finding related to MACS cell sorting procedure) (arrows and inset). **F**-**G**. Day 15; basic (**F**) or DEX (**G**) medium. Cells are larger and more variable in size and shape with more irregular nuclei and more complex cytoplasmic composition. Most cells display specific granules of different size and electron density, suggestive of myeloid differentiation. The arrow in panel F depicts an atypical cell exhibiting a curved nucleus, microvilli, and microvesicular cytoplasmic aggregates (inset). The arrow in panel G depicts an apoptotic cell. **H**. Day 15, DEX medium. Atypical cell showing irregular curved nucleus, localized cytoplasmic aggregate of microvesicular structures, and microvilli, associated with evidence of exocytosis of lamellated material (arrow and inset). **E**-**H**: (uranyl acetate/lead citrate stain; size bar = 2 μm).

By phase contrast microscopy, expanded CB-CD34+ cells appeared round, with increasing variability in cell size after one week in culture (Figure 2 C and D, inset). Occasional pseudopodia-like cellular extensions were noted in all preparations. Giemsa-Wright-stained cytospin preparations allowed a more detailed morphological analysis of expanding CB CD34+ cells. At day 0, freshly isolated CB CD34+ cells appeared uniformly round, relatively small and “lymphocyte-like” with a small amount of cytoplasm and a high nuclear/cytoplasmic ratio (Figure 2A). Short and slender membrane projections were noted. At culture day 3, the cells appeared larger with more abundant and basophilic cytoplasm (Figure 2B). Corresponding to the observed early increase in cell growth rate at this time point, brisk mitotic activity was noted (Figure 2B, arrow). By culture day 15, the cells were significantly more pleomorphic, suggestive of differentiation along divergent hematopoietic pathways (Figure 2C-D). The cells displayed distinct basophilic and eosinophilic tinctorial qualities, associated with increased granularity and vacuolization of the often voluminous cytoplasm (Figure 2C-D). The cellular morphology of most cells was suggestive of myeloblast/early promyelocyte differentiation, although lymphoid or other lineage differentiation cannot be reliably distinguished in these preparations based on morphology alone. The morphology of cells cultured in DEX medium appeared similar to that of cells cultured in basic medium (Figure 2C-D).

We then studied the ultrastructural features of freshly isolated CB-CD34^+^ cells and expanded CB-CD34^+^ cells after two weeks in culture. Freshly isolated CB-CD34+ cells appeared uniform in size and shape and resembled large lymphocytes with a cell diameter ranging between 3.3 μm and 5.0 μm (average diameter: 4.29 ± 0.15 μm), a high nuclear-cytoplasmic ratio, few cytoplasmic organelles, and short, slender membrane projections (Figure 2E). Occasionally, electron dense granules measuring about 50 nm in diameter were noted adjacent to the cells or in submembranous vacuoles, consistent with residual, partially endocytosed immunomagnetic beads utilized for MACS sorting of the cells (Figure 2E, arrows).

After two weeks in culture, the cells were found to be significantly larger and more heterogeneous in size and cellular composition (Figure 2F-H). Cells cultured in DEX media were larger than those cultured in basic media (average diameter: 9.35 ± 1.82 μm versus 7.30 ± 1.90 μm; *P* < 0.0001). The vast majority of cells displayed evidence of hematopoietic differentiation along myeloid, erythroid and other lineages, recognizable by their specific cytoplasmic granules. In this background of hematopoietic progenitor cells, scattered cells were seen with features not readily classifiable according to any of the hematopoietic lineages. These atypical cells showed a large, often curved nucleus, prominent cytoplasmic organelles, villous membrane projections, and/or vesicles with occasional rudimentary electron-dense lamellar structures reminiscent of prelamellar bodies of alveolar type II cells (Figures 2F and 2H, arrows). Occasionally, these rudimentary lamellated structures were present in perimembranous location or attached to the outer cell membrane, suggestive of exocytosis (Figure 2H, arrows). These atypical cells did not contain the specific granules characteristic of hematopoietic precursors. Conversely, villous membrane projections and lamellated cytoplasmic organelles, as seen in these atypical cells, were not seen in identifiable myeloid or erythroid precursors.

### Analysis of respiratory epithelial gene expression of expanded CB-CD34+ cells

**Figure 3:**
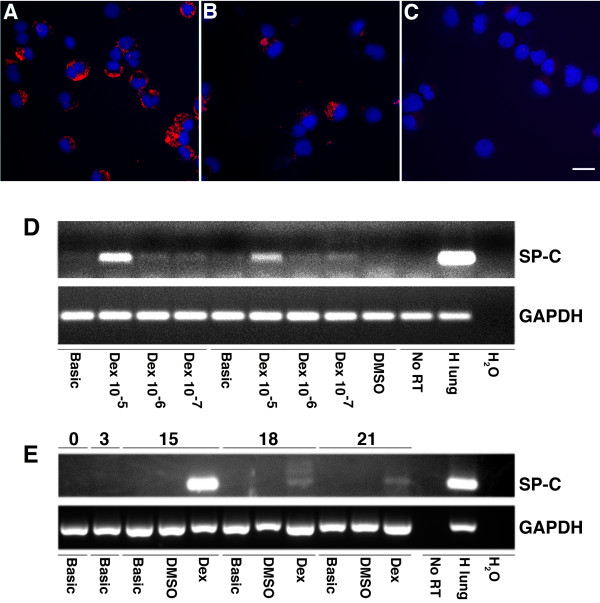
**Analysis of respiratory epithelial cell-like differentiation in expanded CB-CD34+ cells. A**-**C**: Immunofluorescence analysis of surfactant protein-C expression in expanded CB-CD34+ cells, day 21, showing SP-C immunoreactivity (red) in cells cultured in DEX medium (**A**) and basic medium (**B**). **C**: Isotype IgG control. DAPI counterstain (blue). Scale bar = 20 μm. **D**. RT-PCR analysis of surfactant protein-C gene expression in expanded CB-CD34+ cells exposed to Dex at concentrations ranging from 10^-7^M to 10^-5^M. Shown are the results of two representative isolates. Highest SP-C expression of SP-C (327 bp) is seen in the presence of Dex at 10^-5^M. Positive (human lung) and negative controls (omission reverse transcriptase, H_2_O control) were included. The housekeeping gene GAPDH (407 bp) was included as loading control. **E**: RT-PCR analysis of surfactant protein-C gene expression in expanded CB-CD34+ cells. Shown are the results of one representative isolate. Expression of SP-C is seen in the presence of dexamethasone (DEX, 10^-5^M) after 15, 18 and 21 days of culture. Controls were as described under **D**.

Immunofluorescence analysis of cytospin preparations of expanded CB-CD34+ cells examined after 3 weeks in culture revealed the presence of granular cytoplasmic SP-C immunoreactive material in scattered cells (Figure 3A-B). Although the proportion of cells exhibiting SP-C immunoreactivity was insufficient to allow formal quantitation (<5% of total cells), their number appeared higher in DEX-expanded preparations than in basic medium-expanded preparations. Immunoreactivity was absent in preparations exposed to isotype IgG control (Figure 3C).

Respiratory epithelial cell-like differentiation of expanded CB-CD34+ cells was further assessed by semi-quantitative RT-PCR analysis of SP-C gene expression. Three isolates were incubated in DEX medium with DEX concentrations ranging from 10^-7^M to 10^-5^M or basic medium. SP-C expression was observed in the presence of DEX in all three isolates, with highest levels of expression in association with DEX at 10^-5^M in 2/3 cases (Figure 3D) and in association with DEX at 10^-6^M in the third case. We then studied SP-C expression in seven randomly selected isolates incubated in basic medium or DEX medium (10^-5^ in DMSO). SP-C mRNA expression, suggestive of alveolar epithelial type II cell differentiation, was observed in 6/7 DEX-exposed CD34+ cell isolates and in 2/7 basic medium-exposed isolates. None of the isolates cultured for less than one week showed detectable SP-C expression. Results of a representative isolate are shown in Figure 3E.

### Analysis of engraftment of expanded CB-CD34+ cells in lungs of newborn mice

The collective results from the previous morphologic and gene expression studies suggest that culture of CB-CD34+ cells in optimal conditions, achieved by exposure to KGF, retinoic acid and dexamethasone, can induce alveolar epithelial type II cell-like differentiation *ex vivo*. In the second part of this study, we investigated whether expanding CB-CD34+ cells maintain the previously demonstrated potential of freshly isolated CB-CD34+ cells to engraft and transdifferentiate to respiratory epithelial cells *in vivo*.

**Figure 4:**
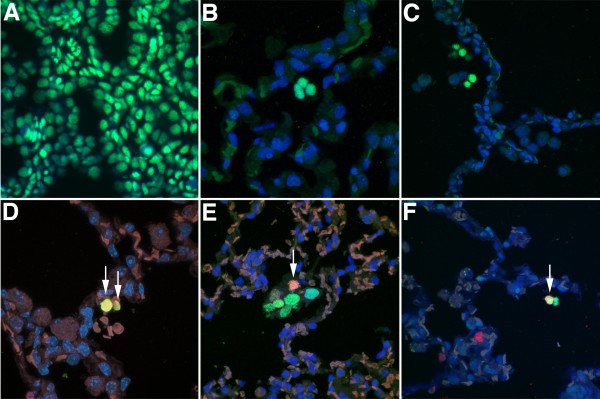
**Analysis of engraftment and proliferation of CB-CD34-derived cells. A-C**: Alu-FISH analysis. **A**: Human postmortem lung tissue (positive control) showing nuclear positivity in all cells. **B**: Representative micrograph of lungs of animal treated with CD34+ cells expanded in basic medium showing a cluster of alu-FISH-positive cells within airspace. **C**. Lung of animal treated with DEX-expanded cells showing collection of alu-FISH-positive cells along the alveolar wall, admixed with larger-sized, FISH-negative murine alveolar macrophages. **A**-**C**: alu-FISH analysis (green) with DAPI counterstain (blue). **D-F**: Alu-FISH analysis combined with anti-Ki67 immunofluorescence. **D**. Representative micrograph of animal treated with freshly isolated CD34+ cells, showing relatively large-sized proliferating alu-FISH positive cells within the alveolar wall (arrows). **E**. Lungs of animal treated with CD34+ cells expanded in basic medium showing a large cluster of alu-FISH-positive cells free within the airspace. Proliferative activity is present in these CD34-derived cells (arrow). **F**. Lungs of animal treated with DEX-expanded CD34+ cells, similarly showing Ki67-positivity in FISH-positive intraalveolar cells (arrow). Several proliferating FISH-negative murine cells are noted. **D**-**F**: alu-FISH analysis (green) combined with anti-Ki67 immunofluorescence (red); DAPI counterstain (blue).

Engraftment was studied 8 weeks after intranasal inoculation of cultured CB-CD34+ cells by FISH analysis for human alu sequences [7]. Alu-FISH-positive cells were readily identified in all recipient animals, although the recovery rates, cellular location, and morphologic appearances varied widely between the different study groups. In recipients of expanded CB-CD34+ cells (basic or DEX) virtually all alu-FISH-positive nuclei were free-floating in the airspaces, often in aggregates of cells with homogeneous, small-sized nuclei (Figure 4B-C). In animals treated with freshly isolated CB-CD34+ cells, in contrast, the vast majority of FISH-positive nuclei was relatively large-sized (equivalent in size to neighboring alveolar epithelial cell nuclei) and localized to the alveolar wall (Figure 4D). Although the number of epithelial-appearing alu-FISH-positive nuclei observed was small in all groups, the amount of engrafted epithelial-like cells appeared much lower following administration of expanded cells than after administration of freshly isolated CB-CD34+ cells.

#### Analysis of proliferation

Proliferation of engrafted cord blood-derived cells was assessed by combining human-specific alu-FISH analysis with anti-Ki67 immunofluorescence staining. In concordance with our previous observations [7], proliferative activity could be observed in a small fraction (<5%) of cord blood-derived type II-cell-like engrafted cells in animals treated with freshly isolated CD34+ cells (Figure 4D). In animals treated with expanded cells, proliferative activity in cord-blood-derived cells was overall more prevalent, and readily visualized in single or clustered lymphoid/myeloid-like mononuclear cells within the airspaces (Figure 4E-F).

### Analysis of cell fate of expanded CB-CD34+ cells in lungs of newborn mice

#### Analysis of respiratory epithelial differentiation

**Figure 5:**
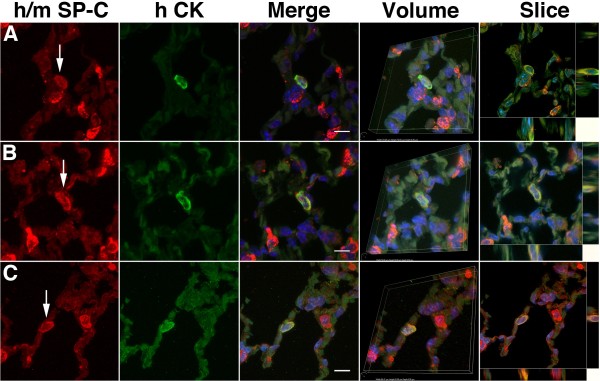
**Analysis of alveolar epithelial cell-like transdifferentiation of CB-CD34-derived cells.** Confocal fluorescence microscopy of lungs subjected to combined anti-human-cytokeratin (green) and anti-mouse/human SP-C (red) immunofluorescence staining. **A**-**B**: Representative micrograph of lungs of animal treated with freshly isolated CD34+ cells. SP-C-positive human-derived epithelial cells were readily visualized (arrows). **C**: Similar double immunofluorescence staining showing one exceedingly rare SP-C-immunoreactive human-derived cell (arrow) observed in an animal treated with expanded cells. **A**-**C**. Merging of the images shows colocalization of granular SP-C-positive material in the CK-positive, human-derived epithelial cells. The SP-C-positive material is coarsely granular, consistent with surfactant-containing lamellar bodies. Colocalization persists at all three-dimensional volume angles analyzed. Volume slices along the *xz* (horizontal) and *yz* (vertical) axes both show colocalization of green and red signals in the same cell, resulting in a yellow-orange composite signal and confirming the unequivocal presence of SP-C-positive material in this human CK-positive, human cord blood-derived epithelial cell. **A**-**C**: Anti-human cytokeratin stain (green) combined with anti-human/mouse SP-C stain (red), DAPI counterstain. Scale bar = 10 μm.

To determine the capacity of CB-CD34+ cells to undergo respiratory epithelial differentiation, we performed double immunofluorescence labeling studies combining anti-human cytokeratin staining (as marker of human-derived epithelial cells) with SP-C staining (as marker of respiratory epithelial cells). In all study groups, rare cells exhibiting colocalization of human cytokeratin and SP-C could be identified, suggestive of transdifferentiation of cord blood-derived cells to surfactant-producing alveolar epithelial cells (Figure 5). Cytokeratin immunoreactivity was absent in surrounding murine alveolar epithelial type II cells, confirming the human-specificity of the anti-keratin antibody. Three-dimensional volume reconstruction and volume slicing with x/y/z axis analysis confirmed unequivocal localization of the SP-C-positive material within the cells (Figure 5). These cord blood-derived type II-like cells were sparse in all groups, but were in particular exceedingly rare in animals treated with expanded (DEX or basic) cells (0.32 ± 0.35 and 0.25 ± 0.38 cells per 10 40x high-power fields, respectively; based on four animals per group) compared with those treated with freshly isolated cells (0.62 ± 0.53 per 10 40x high-power fields, difference not statistically significant).

#### Analysis of leukocyte differentiation

**Figure 6:**
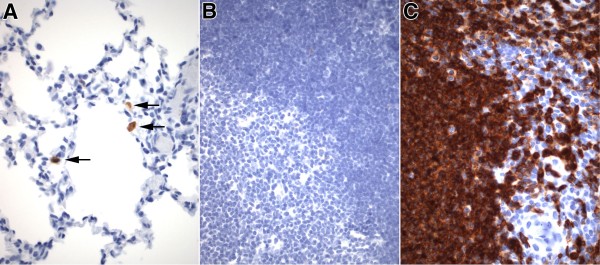
**Analysis of leukocyte differentiation of CB-CD34+ cells.** Immunohistochemical analysis of leukocyte differentiation using a human-specific anti-CD45 (leukocyte common antigen) antibody. **A**. Representative micrograph of lungs of animal treated with DEX-expanded CB-CD34+ cells at post-inoculation week 8. Scattered CD45-positive mononuclear cells are noted along the alveolar wall. CD45-immunoreactivity is absent in murine thymic lymphocytes (negative control) (**B**) and diffusely positive in human lymphocytes (**C**, human tonsil, positive control), confirming the human-specific nature of the antibody. **A**-**C**: avidin-biotin peroxidase staining, hematoxylin counterstain. Original magnification: X400.

The alu-FISH-positive cells detected as single or clustered cells within the airspaces of animals treated with expanded cells were morphologically suggestive of mononuclear leukocytes. Lung sections were studied by immunohistochemical analysis using an antibody specific for the human CD45 antigen (LCA, leukocyte common antigen). Scattered cord blood-derived CD45-positive mononuclear cells, consistent with lymphoid cells or monocytes/macrophages were identified within the airspaces or along the alveolar wall (Figure 6A). Absence of CD45 immunoreactivity in murine thymic lymphocytes (Figure 6B) and strong immunoreactivity in human tonsillar lymphocytes (Figure 6C) confirmed the specificity of the antibody for CD45 antigen of human origin. The combined immunohistochemical results indicate that a significant fraction of CD34-derived cells persist as intraalveolar non-epithelial cells, including leukocytes.

### Analysis of effect of expanded CB-CD34+ cells on lung growth and alveolarization

The lung volume, V(lu), was larger in animals treated with CD34+ cells expanded in basic media than in sham controls (*P* < 0.05). A similar trend was seen in animals treated with DEX-expanded cells (Table 1). Computer-assisted stereological volumetry was applied to determine the relative contributions of the various lung compartments (peripheral air-exchanging parenchyma versus airspace) to the total lung volume. The areal density of air-exchanging parenchyma, A_A_(ae/lu), which represents the parenchymal tissue fraction, tended to be larger in animals treated with DEX-expanded CD34+ cells than in the other groups. The total volume of air-exchanging parenchyma, V(ae), which takes into account both V(lu) and A_A_(ae/lu), was significantly 25% larger in animals with CD34+ cells expanded in basic media (*P* < 0.02) and 30% larger in animals with DEX-expanded CD34+ cells (*P* < 0.05) than in sham controls. Similarly, V(ae) normalized to body weight [V(ae)/BW] was significantly larger in animals treated with both types of expanded CD34+ cells compared with sham controls (*P* < 0.01). In contrast, treatment with fresh CD34+ cells had no effect on V(ae) or V(ae)/BW (Table 1).

**Table 1:** Biometry and volumetry data

	**PBS (8)**	**CD34+ Fresh (3)**	**CD34+ Basic (12)**	**CD34+ Dex (8)**
**Body wt (g)**	26.37 ± 2.76	21.00 ± 2.95*	25.50 ± 4.43	23.01 ± 3.29*
**V(lu) (μl)**	669 ± 161	595 ± 105	814 ± 153*	738 ± 113
**A**_**A**_**(ae/lu)**	21.3 ± 4.7	20.2 ± 3.0	21.1 ± 3.1	24.0 ± 4.9
**V(ae) (μl)**	136 ± 23	118 ± 12	170 ± 31*	177 ± 43*
**V(ae)/BW (μl/g)**	5.33 ± 0.48	5.69 ± 0.82	6.84 ± 1.35**	7.45 ± 1.92**

Animals were treated with freshly isolated CB-CD34+ cells (0.5 x 10^6^ cells/pup) or CB-CD34+ cells expanded in basic medium or DEX medium (1-2 x 10^6^ cells/pup). Sham controls received equal volume PBS.

Values represent mean ± SD of (n) animals per group.

V(lu): lung volume; A_A_(ae/lu): areal fraction of air-exchanging parenchyma; V(ae): volume of air-exchanging parenchyma.

*: *P* < 0.05; **: *P* < 0.01 versus PBS-treated sham controls.

(Student-t test).

**Figure 7:**
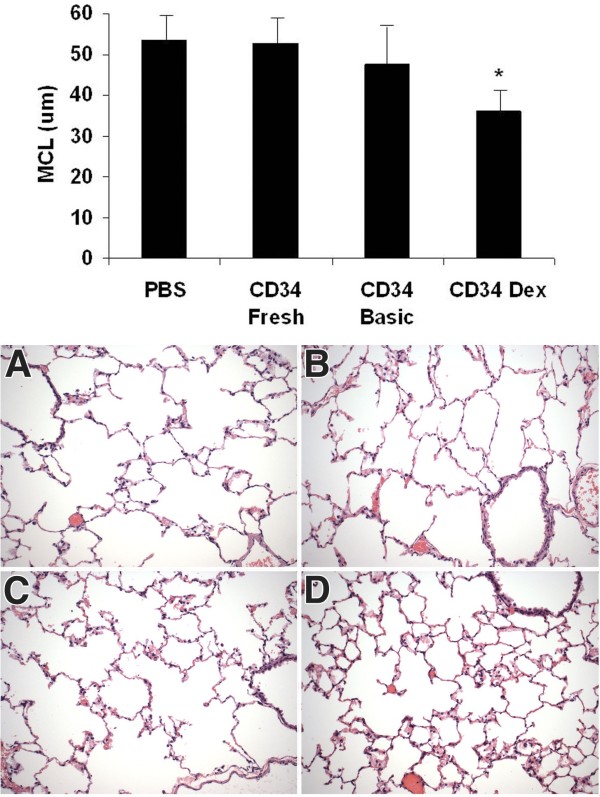
**Analysis of alveolarization.** Top. Morphometric analysis of mean cord length (MCL). Values represent mean ± SD of at least 3 animals per group. *: *P* < 0.01 versus PBS-treated sham controls (ANOVA with post hoc Scheffe test). Bottom. Representative micrographs showing lung parenchyma of animals treated with PBS (sham, **A**), fresh CB-CD34+ cells (**B**), CB-CD34+ cells expanded in basic medium (**C**) or CB-CD34+ cells expanded in DEX medium (**D**), 8 weeks post inoculation. Animals treated with DEX-expanded CD34+ cells (**D**) showed more advanced alveolar architectural remodeling with more complex alveolar septation than animals of the other study groups (**A**-**C**). Hematoxylin-eosin staining, original magnification: X200.

The lung histology of Dox-treated double transgenic CCSP+/FasL + animals treated with CB-CD34+ cells expanded in basic media appeared similar to that of animals treated with freshly isolated CB-CD34+ cells, and sham (PBS) controls (Figure 7 A-C). In these three groups, the airspaces were markedly dilated and simplified, resulting in an emphysematous appearance (Figure 7 A-C). Lungs of double transgenic animals treated with DEX-expanded cells showed smaller airspaces and a more complex alveolar network (Figure 7 D). Small numbers of scattered alveolar macrophages, were seen in all groups and were usually limited to the subpleural regions (not shown). There was no other histologic evidence of interstitial or intraalveolar inflammation in any of these groups.

The degree of alveolarization was estimated morphometrically by determination of the mean cord length (MCL) (Figure 7, top). The MCL was significantly 48% smaller in animals treated with DEX-expanded CD34+ cells than in sham controls (36.1 ± 7.0 μm versus 53.5 ± 9.5 μm, *P* < 0.001), indicative of significantly enhanced alveolar septation in animals treated with DEX-exposed CD34+ cells. The MCL of animals treated with CD34+ cells expanded in basic medium was similar to that of animals treated with fresh CD34+ cells or sham animals (Figure 7, top).

## Discussion

We recently reported that freshly isolated and intranasally delivered cord blood-derived CD-34+ cells (CB-CD34+) are capable of stable engraftment, clonogenic expansion, and respiratory epithelial transdifferentiation in injured newborn lungs [7]. In the present study, we investigated the therapeutic potential of *ex vivo* expanded CB-CD34+ cells in injured newborn lungs. Our first aim was to determine whether expanding CB-CD34+ cells can be induced to go undergo respiratory epithelial differentiation in culture. When exposed to conditions conducive to respiratory epithelial differentiation, including the appropriate cocktail of cytokines, growth factors, and glucocorticoids (dexamethasone, DEX), CB-CD34+ cells demonstrated an innate potential to undergo respiratory epithelial cell-like differentiation, as evidenced by SP-C mRNA expression in virtually all DEX-exposed cell isolates examined. Respiratory epithelial cell-like transdifferentiation of a subset of DEX-expanded cells was confirmed by focal cytoplasmic presence of immunoreactive SP-C protein, as well as ultrastructural features reminiscent of alveolar type II cell-like differentiation, such as prelamellar body-like organelles, microvilli, and apparent exocytosis of lamellated electron dense material. The effect of Dex exposure on SP-C expression was studied for concentrations ranging between 10^-5^ and 10^-7^M (100-10.000 nM). In this limited dose response study, SP-C expression was highest in cells exposed to 10^-5^M/10^-6^M Dex. As a reference, the Dex concentration used for *ex vivo* stem cell and/or erythroid progenitor cell expansion in various reported experimental or clinical models ranges around 10^-6^M [26-28]. The customary Dex concentration used to study human alveolar type II cell differentiation *in vitro* is considerably lower (10nM, 10^-8^M) [17].

Sueblinvong et al. [29] previously reported respiratory epithelial gene expression, including SP-C expression, in human cord blood-derived mesenchymal stem cells cultured in specialized airway growth media and/or specific growth factors such as KGF and retinoic acid. Similarly, Berger et al. [30,31] reported respiratory epithelial differentiation in cord blood-derived multilineage progenitor cells cultured and differentiated in standard mesenchymal stem cell growth medium and small airway growth medium (SAGM), respectively. To our knowledge, this is the first study to demonstrate that cord blood-derived CD34+ cells, as well, have the capacity to undergo *in vitro* respiratory epithelial cell-like differentiation when exposed to the appropriate differentiation agents. Of note, the current *in vitro* studies demonstrate that cord blood-derived CD34+ cells are capable of development of a type II cell-like phenotype in the absence of respiratory epithelial cells or their cell components (such as vesicles) and thus confirm our prior *in vivo* observation [7] that transdifferentiation of CB-CD34+ cells to type II cell-like cells can occur by fusion-independent mechanisms.

The second aim of this study was to determine whether CB-CD34+ cells, briefly expanded *ex vivo*, have the capacity to engraft and improve alveolar remodeling in injured newborn murine lungs *in vivo*. Consistent with our belief that direct intrapulmonary delivery of stem cells represents a biologically sound and clinically relevant strategy for restoration of injured respiratory epithelium, we chose the intranasal/intrapulmonary, rather than systemic route of administration for delivery of stem cells in this study, as described in prior studies [7,18]. The cell fate and effects of CB-CD34+ cells expanded *ex vivo* in basic or DEX media were compared with those of freshly isolated CB-CD34+ cells.

In agreement with our previous study [7], intranasal delivery of freshly isolated CD34+ cells led to modest but stable engraftment of SP-C immunoreactive, transdifferentiated alveolar type II cell-like cells. Engraftment in the form of transdifferentiated alveolar epithelial-like cells was negligible following delivery of expanded CD34-derived cells, whether cultured in DEX or basic media. Instead, the vast majority of expanded cells or their progeny were recovered as aggregates of non-epithelial, at least partly leukocytic (lymphoid and/or myeloid) mononuclear cells free-floating within the airspaces. It needs to be stressed that studies based on detection of human-specific markers in homogenized lung tissues may have concluded that inoculation of expanded CD34+ results in massive “engraftment” of cord blood-derived cells. However, the term engraftment in the strictest sense may be better reserved for unequivocal stable integration of donor-derived cells within the alveolar wall, preferably with demonstrated evidence of respiratory epithelial transdifferentiation and clonogenic expansion.

Administration of expanded cells, whether grown in basic or DEX media, was associated with significant lung growth at 8 weeks post-transplantation, as determined by stereological volumetry of the air-exchanging parenchyma. The lung growth was associated with enhanced alveolar septation in animals treated with DEX-expanded cells, but not in those treated with cells expanded in basic medium in the absence of DEX. Administration of freshly isolated CB-CD34+ cells, while resulting in more effective engraftment and transdifferentiation, did not affect either lung growth or alveolar development. While the capacity of DEX-expanded cells to promote lung growth and alveolar remodeling in injured newborn lungs is highly encouraging, the mechanisms underlying these beneficial effects remain unclear.

In view of the negligible engraftment and transdifferentiation rates achieved with expanded cells, it is unlikely that the positive impact is related to regeneration of injured alveolar epithelium by transdifferentiated type II cell-like cells. Rather, it is plausible that the growth-promoting effects of DEX-expanded cells are linked to paracrine effects related to factors produced by the observed relatively abundant cord blood-derived intraalveolar mononuclear cells. By analogy, the beneficial effects of a variety of stem or progenitor cells types in a wide range of lung injury models have mostly been attributed to cell-derived paracrine factors, as lung engraftment of stem or progenitor cells has been reported as much less than 5% in the majority of studies (reviewed in [5]). Paracrine mechanisms of action have been studied most extensively in mesenchymal stem cells. The beneficial effects of stem cell-derived paracrine factors, such as interleukin (IL)-10, IL1ra, KGF, prostaglandin E2, tumor necrosis factor-α stimulated gene/protein 6, and others, have been linked to their anti-inflammatory, immunomodulatory and/or growth promoting functions (reviewed in [32]).

It needs to be emphasized that inherent limitations of our experimental model, which is based on intranasal stem cell delivery to an immunocompetent host, may lead to underestimation of the full therapeutic potential of cord blood-derived stem cell therapy in the clinical context. The intranasal cell administration method is *inevitably* inefficient: a significant and highly variable proportion of intranasally inoculated cells are lost by spillage through mouth or gastrointestinal tract. If translation of this preclinical study to the newborn context becomes a reality, preterm newborns are expected to receive autologous stem cells via an endotracheal tube, which will eliminate the two major sources of graft loss in our experimental model.

While the FasL transgenic mouse model is a valid model of neonatal lung injury [19], it is centered on a human-to-mouse xenograft in an immunocompetent mouse strain. As we previously demonstrated, a large fraction of the graft is eliminated by host rejection during the early post-transplant period, [7,18]. However, in concordance with other groups who demonstrated similar long-term persistence of human-derived stem cells in immunocompetent rodent (rat or mouse) lungs or other organs [33-35], we were able to detect human-derived cells up to 8 weeks after inoculation. The mechanisms whereby (a subset of) human-derived cells can persist in an immunocompetent rodent host, even in small amounts, remain to be determined. We speculate that at least some of the reported functional or phenotypic mechanisms by which tumor stem cells (TSCs) evade immunosurveillance and immune-mediated rejection may be implicated. Such reported mechanisms include altered immunogenicity of TSCs, production of TSC-derived regulatory molecules, and interaction of TSCs with tumor-infiltrating immune cells (reviewed in [36]).

Other limitations of this study are acknowledged. First, our study was restricted to the use of a single, arbitrarily selected stem cell loading dose per study group. Future dose-response studies will be required to determine the optimal dose of stem cells to achieve functional amelioration of alveolar remodeling for each stem cell type and delivery route studied. Second, the long-term outcome of intranasal stem cell delivery remains unclear. The effects of the long-term chimerism created by the intrapulmonary persistence of actively replicating hematopoietic elements with presumed ongoing paracrine effects certainly deserve future experimental attention. Third, analysis of respiratory epithelial differentiation, both *ex vivo* and *in vivo*, was mainly focused on surfactant protein-C expression as marker of alveolar type II cell differentiation. Whether the respiratory epithelial-like cells derived from CB-CD34+ cells have additional morphologic, biochemical or functional characteristics of alveolar epithelial cells remains unclear. Fourth, the effects of stem cell delivery on alveolar remodeling were limited to morphometric assessment of alveolar septation. While mean cord length (MCL) is an accepted measure of alveolar remodeling in newborn lungs, and increased alveolar septation is a highly encouraging predictor of ameliorated functional outcome, future studies will be needed to establish the functional effects of stem cell therapy. Fifth, this study only investigated the fate and effects of *term* CB-CD34+ cells. The characteristics of preterm cells, highly relevant in translational context of preterm lung injury, remain undetermined. Finally, this study was limited to the use of CD34+ hematopoietic progenitor cells. The use of other cord blood-derived stem cells, in particular mesenchymal stem cells or their secreted components, alone or in combination with CD34+ cells, needs to be explored.

## Conclusions

Our results suggest that cord blood-derived CD34+ cells, when cultured in the presence of growth factors, cytokines, retinoic acid and glucocorticoids, have the (limited) potential to undergo fusion-independent differentiation to alveolar epithelial cell-like cells *in vitro*. *Ex vivo* expanded CD34+ cells appear to lose their engraftment potential following intrapulmonary delivery *in vivo*; instead, they tend to persist long-term as an actively replicating population of intraalveolar mononuclear cells. By currently undetermined mechanisms, intrapulmonary delivery of DEX-expanded cells promotes growth and alveolar remodeling of injured newborn lungs. The suggestion that expanded cord blood-derived CD34+ cells are capable of rescuing alveolar development in injured newborn lungs, at least in part, opens exciting avenues for future preclinical studies. Such studies will need to focus on the refining of *ex vivo* expansion techniques to determine the delicate balance between cell growth, retention of critical stemness characteristics of CD34+ cells (i.e. potential for self renewal and engraftment), and appropriate lineage selection.

## Competing interests

ME De Paepe is applying for a patent relating to lung regeneration using cord blood-derived hematopoietic stem cells. ME De Paepe is currently funded, in part, by a research grant from Perkin-Elmer/Viacord (the present study and/or manuscript were not financed by Perkin Elmer/Viacord). The other authors declare that they have no competing interests.

## Authors’ contributions

QM participated in the design of the study and carried out the in vitro and in vivo experiments, SC participated in the in vivo studies and contributed to the immunohistochemical and molecular analyses, SG participated in the in vitro studies and contributed to the immunohistochemical and molecular analyses, JFP contributed to study design and writing of the manuscript, MEDP conceived of the study, participated in its design and was a major contributor in writing the manuscript. All authors read and approved the final manuscript.

## References

[B1] Jobe AH Bancalari E Bronchopulmonary dysplasia Am J Respir Crit Care Med 2001 163 1723 1729 11401896 10.1164/ajrccm.163.7.2011060

[B2] Husain AN Siddiqui NH Stocker JT Pathology of arrested acinar development in postsurfactant bronchopulmonary dysplasia Hum Pathol 1998 29 710 717 10.1016/S0046-8177(98)90280-5 9670828

[B3] Jobe AJ The new BPD: an arrest of lung development Pediatr Res 1999 46 641 643 10.1203/00006450-199912000-00007 10590017

[B4] De Paepe ME Mao Q Powell J Rubin SE DeKoninck P Appel N Dixon M Gundogan F Growth of pulmonary microvasculature in ventilated preterm infants Am J Respir Crit Care Med 2006 173 204 211 16210670 10.1164/rccm.200506-927OC PMC2662989

[B5] Alphonse RS Rajabali S Thebaud B Lung injury in preterm neonates: the role and therapeutic potential of stem cells Antioxid Redox Signal 2012 17 1013 1040 10.1089/ars.2011.4267 22400813

[B6] O’Reilly M Thebaud B Cell-based strategies to reconstitute lung function in infants with severe bronchopulmonary dysplasia Clin Perinatol 2012 39 703 725 10.1016/j.clp.2012.06.009 22954277 PMC7112346

[B7] De Paepe ME Mao Q Ghanta S Hovanesian V Padbury JF Alveolar epithelial cell therapy with human cord blood-derived hematopoietic progenitor cells Am J Pathol 2011 178 1329 1339 10.1016/j.ajpath.2010.11.062 21356383 PMC3128500

[B8] Ikegami M Jobe AH Huffman Reed JA Whitsett JA Surfactant metabolic consequences of overexpression of GM-CSF in the epithelium of GM-CSF-deficient mice Am J Physiol 1997 273 L709 L714 9357844 10.1152/ajplung.1997.273.4.L709

[B9] Pelaez A Bechara RI Joshi PC Brown LA Guidot DM Granulocyte/macrophage colony-stimulating factor treatment improves alveolar epithelial barrier function in alcoholic rat lung Am J Physiol Lung Cell Mol Physiol 2004 286 L106 L111 14504066 10.1152/ajplung.00148.2003

[B10] Baytur YB Ozbilgin K Yuksel H Kose C Antenatal administration of granulocyte-macrophage colony-stimulating factor increases fetal lung maturation and endothelial nitric oxide synthase expression in the fetal rat lung Eur J Obstet Gynecol Reprod Biol 2008 136 171 177 10.1016/j.ejogrb.2007.03.010 17478029

[B11] Massaro D De Carlo Massaro G Retinoids, alveolus formation, and alveolar deficiency: clinical implications Am J Respir Cell Mol Biol 2003 28 271 274 10.1165/rcmb.F263 12594052

[B12] Chytil F Retinoids in lung development FASEB J 1996 10 986 992 8801181 10.1096/fasebj.10.9.8801181

[B13] Ware LB Matthay MA Keratinocyte and hepatocyte growth factors in the lung: roles in lung development, inflammation, and repair Am J Physiol Lung Cell Mol Physiol 2002 282 L924 L940 11943656 10.1152/ajplung.00439.2001

[B14] Sugahara K Rubin JS Mason RJ Aronsen EL Shannon JM Keratinocyte growth factor increases mRNAs for SP-A and SP-B in adult rat alveolar type II cells in culture Am J Physiol 1995 269 L344 L350 7573468 10.1152/ajplung.1995.269.3.L344

[B15] Deterding RR Jacoby CR Shannon JM Acidic fibroblast growth factor and keratinocyte growth factor stimulate fetal rat pulmonary epithelial growth Am J Physiol 1996 271 L495 L505 8897895 10.1152/ajplung.1996.271.4.L495

[B16] Mason RJ Lewis MC Edeen KE McCormick-Shannon K Nielsen LD Shannon JM Maintenance of surfactant protein A and D secretion by rat alveolar type II cells in vitro Am J Physiol Lung Cell Mol Physiol 2002 282 L249 L258 11792629 10.1152/ajplung.00027.2001

[B17] Gonzales LW Guttentag SH Wade KC Postle AD Ballard PL Differentiation of human pulmonary type II cells in vitro by glucocorticoid plus cAMP Am J Physiol Lung Cell Mol Physiol 2002 283 L940 L951 12376347 10.1152/ajplung.00127.2002

[B18] Fritzell JA Jr Mao Q Gundavarapu S Pasquariello T Aliotta JM Ayala A Padbury JF De Paepe ME Fate and effects of adult bone marrow cells in lungs of normoxic and hyperoxic newborn mice Am J Respir Cell Mol Biol 2009 40 575 587 10.1165/rcmb.2008-0176OC 18988921 PMC2677437

[B19] De Paepe ME Gundavarapu S Tantravahi U Pepperell JR Haley SA Luks FI Mao Q Fas-ligand-induced apoptosis of respiratory epithelial cells causes disruption of postcanalicular alveolar development Am J Pathol 2008 173 42 56 10.2353/ajpath.2008.071123 18535181 PMC2438284

[B20] De Paepe ME Haley SA Lacourse Z Mao Q Effects of Fas-ligand overexpression on alveolar type II cell growth kinetics in perinatal murine lungs Pediatr Res 2010 68 57 62 10.1203/PDR.0b013e3181e084af 20375852 PMC2888646

[B21] De Paepe ME Mao Q Chao Y Powell JL Rubin LP Sharma S Hyperoxia-induced apoptosis and Fas/FasL expression in lung epithelial cells Am J Physiol Lung Cell Mol Physiol 2005 289 L647 L659 10.1152/ajplung.00445.2004 16148053

[B22] Tichelaar JW Lu W Whitsett JA Conditional expression of fibroblast growth factor-7 in the developing and mature lung J Biol Chem 2000 275 11858 11864 10.1074/jbc.275.16.11858 10766812

[B23] De Paepe ME Johnson BD Papadakis K Luks FI Lung growth response after tracheal occlusion in fetal rabbits is gestational age-dependent Am J Respir Cell Mol Biol 1999 21 65 76 10385594 10.1165/ajrcmb.21.1.3511

[B24] De Paepe ME Johnson BD Papadakis K Sueishi K Luks FI Temporal pattern of accelerated lung growth after tracheal occlusion in the fetal rabbit Am J Pathol 1998 152 179 190 9422535 PMC1858114

[B25] Aherne WA Dunnill MS Aherne WA, Dunnill MS The estimation of whole organ volume Morphometry 1982 London: Edward Arnold Ltd 10 18

[B26] Migliaccio G Sanchez M Masiello F Tirelli V Varricchio L Whitsett C Migliaccio AR Humanized culture medium for clinical expansion of human erythroblasts Cell Transplant 2010 19 453 469 10.3727/096368909X485049 20149301 PMC4397648

[B27] Ganguli G Back J Sengupta S Wasylyk B The p53 tumour suppressor inhibits glucocorticoid-induced proliferation of erythroid progenitors EMBO Rep 2002 3 569 574 10.1093/embo-reports/kvf114 12034755 PMC1084148

[B28] England SJ McGrath KE Frame JM Palis J Immature erythroblasts with extensive ex vivo self-renewal capacity emerge from the early mammalian fetus Blood 2011 117 2708 2717 10.1182/blood-2010-07-299743 21127173 PMC3062358

[B29] Sueblinvong V Loi R Eisenhauer PL Bernstein IM Suratt BT Spees JL Weiss DJ Derivation of lung epithelium from human cord blood-derived mesenchymal stem cells Am J Respir Crit Care Med 2008 177 701 711 10.1164/rccm.200706-859OC 18063840 PMC2277209

[B30] Berger MJ Minnerath SR Adams SD Tigges BM Sprague SL McKenna DH Jr Gene expression changes with differentiation of cord blood stem cells to respiratory epithelial cells: a preliminary observation Stem Cell Res Ther 2011 2 19 10.1186/scrt60 21489244 PMC3226290

[B31] Berger MJ Adams SD Tigges BM Sprague SL Wang XJ Collins DP McKenna DH Differentiation of umbilical cord blood-derived multilineage progenitor cells into respiratory epithelial cells Cytotherapy 2006 8 480 487 10.1080/14653240600941549 17050253

[B32] Alphonse RS Thebaud B Growth factors, stem cells and bronchopulmonary dysplasia Neonatology 2011 99 326 337 10.1159/000326621 21701205

[B33] Pierro M Ionescu L Montemurro T Vadivel A Weissmann G Oudit G Emery D Bodiga S Eaton F Peault B Short-term, long-term and paracrine effect of human umbilical cord-derived stem cells in lung injury prevention and repair in experimental bronchopulmonary dysplasia Thorax 2012 Epub ahead of print 10.1136/thoraxjnl-2012-202323 23212278

[B34] Chang YS Oh W Choi SJ Sung DK Kim SY Choi EY Kang S Jin HJ Yang YS Park WS Human umbilical cord blood-derived mesenchymal stem cells attenuate hyperoxia-induced lung injury in neonatal rats Cell Transplant 2009 18 869 886 10.3727/096368909X471189 19500472

[B35] Ahn SY Chang YS Kim SY Sung DK Kim ES Rime SY Yu WJ Choi SJ Oh WI Park WS Long-term (postnatal day 70) outcome and safety of intratracheal transplantation of human umbilical cord blood-derived mesenchymal stem cells in neonatal hyperoxic lung injury Yonsei Med J 2013 54 416 424 10.3349/ymj.2013.54.2.416 23364976 PMC3575965

[B36] Qi Y Li RM Kong FM Li H Yu JP Ren XB How do tumor stem cells actively escape from host immunosurveillance? Biochem Biophys Res Commun 2012 420 699 703 10.1016/j.bbrc.2012.03.086 22465008

